# A lack of association between adiponectin polymorphisms and coronary artery disease in a Chinese population

**DOI:** 10.1590/S1415-47572010005000064

**Published:** 2010-09-01

**Authors:** Chen Zhong, Ding Zhen, Qian Qi, Ma Genshan

**Affiliations:** Department of Cardiology, The Affiliated ZhongDa Hospital and Institute of Cardiovascular Disease, Southeast University, NanjingP.R. China

**Keywords:** adiponectin gene, coronary artery disease, single nucleotide polymorphisms

## Abstract

We investigated the association between two single nucleotide polymorphisms (SNPs) in the adiponectin gene (rs822395 and rs266729) and coronary artery disease (CAD) in a case-control study of 198 unrelated Chinese CAD patients (with ≥ 70% coronary stenosis or previous myocardial infarction) and 237 non-CAD controls. The ligase reaction was used to detect SNPs rs822395 and rs266729, and the allelic association of these SNPs with the occurrence and severity of CAD was assessed. There were no significant differences in the genotypic or allelic frequencies of the two SNPs between control and CAD individuals. In addition, there was no association between the two SNPs and the severity of CAD based on the number of diseased vessels. The frequencies of alleles C and G at rs266729 differed significantly between females in the CAD and control groups, but not between males. Female carriers of allele G at rs266729 had a higher risk of CAD compared with allele C carriers (OR = 1.30, 95% CI: 1.09-2.64, p = 0.02). These results indicate a gender-specific effect of the adiponectin gene rs266729 variant in modulating the risk of CAD in women.

## Introduction

Coronary artery disease (CAD) is one of the most common cardiovascular diseases and has a high incidence of morbidity and mortality. CAD is a major public health problem in developing and developed countries and its increasing prevalence is a cause of considerable concern in the medical community worldwide (He *et al.*, 2005). CAD involves genetic and environmental factors and their interaction with each other. Traditional risk factors account for at most one-half of the prevalence of CAD (Wilson *et al.*, 1998; Zdravkovic *et al.*, 2002). Despite attempts to establish the molecular and genetic determinants that could account for variations in CAD (Zdravkovic *et al.*, 2002), the etiology and complex multigenic basis of atherosclerosis is still not completely understood.

Adiponectin is an adipocyte-derived protein, the plasma levels of which have been used as a marker of inflammation and hemostasis. Adiponectin has received considerable attention in recent years because of its insulin-sensitizing and anti-atherogenic activity; indeed, several studies have indicated a gradual decrease in the circulating levels of this protein with increasing insulin resistance (Haluzík *et al.*, 2004; Fisher *et al.*, 2005; Kadowaki and Yamauchi, 2005; Kawano and Arora, 2009). Adiponectin gene activity has an important influence on the susceptibility to insulin resistance, a core determinant of prevalent disorders such as type 2 diabetes mellitus (T2DM), metabolic syndrome and atherosclerosis (Hara *et al.*, 2002; Menzaghi *et al.*, 2002, 2007; Rothenbacher *et al.*, 2005). Various studies have suggested that genetic deficits in adiponectin production or action may contribute to insulin resistance (Filippi *et al.*, 2004), atherosclerosis (Kubota *et al.*, 2002), and inflammation (Ouchi *et al.*, 1999; Kubota *et al.*, 2002; Tilg and Moschen, 2008), all of which are frequently associated with CAD.

In laboratory animals, recombinant adiponectin improves insulin sensitivity, inhibits inflammatory responses and reverses diet-induced lipid abnormalities (Kadowaki and Yamauchi, 2005). Furthermore, when apo E-deficient mice (a model of accelerated atherosclerosis) are crossed with adiponectin transgenic mice the progression of atherosclerosis is inhibited, despite unaltered glucose and lipid metabolism, which suggests that adiponectin has a direct antiatherogenic action (Yamauchi *et al.*, 2003; von Eynatten *et al.*, 2006).

In humans, low levels of adiponectin are associated with an increased risk of cardiovascular disease that includes a characteristic profile of metabolic syndrome, such as a low level of high-density lipoprotein cholesterol (HDL-C), high triglyceride (TG) levels and insulin resistance (Matsubara *et al.*, 2002; Cnop *et al.*, 2003; Ryo *et al.*, 2004; Trujillo and Scherer, 2005). However, both positive (Kumada *et al.*, 2003; Rothenbacher *et al.*, 2005; Otsuka *et al.*, 2006, 2007; Selcuk *et al.*, 2008) and negative (Sattar *et al.*, 2006; Kuller *et al.*, 2007; Gui *et al.*, 2008; Rizza *et al.*, 2009; Karakas *et al.*, 2010) correlations between adiponectin levels and CAD have been reported. In particular, the relationship between common adiponectin variants (rs266729, -11365C > G; rs822395, -4034A > C; rs822396, -3964A > G; rs2241766, +45T > G; and rs1501299, +276G > T) and CAD is inconsistent and unconvincing (Filippi *et al.*, 2005; Pischon *et al.*, 2007).

Although the identification of genetic variants of adiponectin and their relationship with CAD are interesting and promising, the controversial and often inconclusive findings of most studies suggest that the data may be more relevant if obtained from specific ancestral groups. In this regard, there is little information on the relationship between two common SNPs in the adiponectin gene (rs266729, -11365C > G; rs822395, -4034A > C) and the risk of CAD in Chinese populations. In the present study, we examined the relationship between these two SNPs and the risk of CAD in Chinese patients and assessed whether the presence of these SNPs correlated with the severity of coronary lesions.

## Methods

###  Subjects and study groups

From January 2003 to December 2008, 435 unrelated subjects (men 31-86 years old and women 39-83 years old) who were scheduled for elective coronary angiography (CAG) for chest discomfort or suspected CAD were enrolled in the study. The CAD group consisted of 198 patients with documented CAD who had ≥ 70% stenosis in at least one main coronary artery or had experienced a myocardial infarction defined according to World Health Organization criteria. The control group consisted of 237 subjects with no detectable coronary stenosis in CAG. All patients with congenital heart disease, syndrome X, severe liver or kidney disease, non-coronary artery thrombotic disease, or a contraindication for heparin were excluded from the study. Written informed consent was obtained from all participants before enrolling. The study protocol was approved by the Medical Ethics Committee of the Affiliated ZhongDa Hospital of Southeast University.

###  Coronary angiography

All participants underwent elective CAG by the Judkins technique, with images recorded on compact disks for later analysis. The CAD cases were grouped according to the number of significantly stenosed vessels, *i.e.*, individuals with one, two or three affected vessels. Two cardiologists unaware of the impact of their consensus opinion on this study assessed the grade of coronary stenosis. All participants were from the same geographical area and had a similar socioeconomic and ethnic background. Patients without coronary stenosis served as controls.

###  Determination of biochemical and clinical risk factors

At the time of enrollment, data were collected from each subject, including a complete history of cardiovascular risk factors such as hypertension, T2DM and smoking habit. Anthropometric and blood pressure measurements were made according to standard protocols. Hypertension was defined as blood pressure ≥ 140/90 mmHg or the use of antihypertensive medications. Subjects with a history of T2DM, those receiving anti-diabetic medications, and those with a confirmed fasting blood sugar (FBS) concentration > 126 mg/dL (7.0 mmol/L) were considered to have T2DM. Subjects who smoked at least one cigarette per day at the time of enrollment were considered as smokers, as were those who had smoked in the month before the study. The body mass index (BMI) was calculated as weight in kilograms divided by the square of the height in meters (kg/m^2^).

Venous blood samples for lipoprotein and FBS measurements were collected into tubes containing 0.1% EDTA after a 12 h fast. Plasma concentrations of total cholesterol (TC), TG, HDL-C, low-density lipoprotein cholesterol (LDL-C), apolipoprotein A1 (apo A1) and apolipoprotein B (apo B) were determined by standard biochemical methods using a chemical analyzer (Beckman Coulter Synchron Clinical System LX20, Fullerton, CA, USA). Plasma insulin concentrations were measured by a commercial radioimmunoassay kit (Beifang Biotechnical Institute, Beijing, China) with an inter-assay coefficient of variation (CV) of 15% and intra-assay CV of 10%. TG was measured directly when > 4.52 mmol/L (400 mg/dL). All of the plasma samples were thawed only once.

###  DNA extraction and adiponectin genotyping

Genomic DNA was extracted from blood leucocytes by using a whole-blood genomic DNA extraction kit (Axygene Biotechnology Ltd., Hangzhou, China), according to the manufacturer's instructions. The two SNPs in rs822395 and rs266729 were genotyped by the ligase detection reaction (Favis *et al.*, 2000; Xiao *et al.*, 2006) with TaqMan genotyping assays in an ABI Prism 377 sequence detection system (Applied Biosystems, Foster City, CA). Random duplicate samples and sequencing technique were used for quality control in the genotyping. The overall success rate for genotyping was > 96%.

###  Statistical analysis

Continuous data were expressed as the mean ± SD and Students *t*-test was used to analyze differences between the two study groups. Categorical variables between the two groups were compared by the chi-square test and the genotype groups were compared with one-way ANOVA or the chi-square test. The latter test was also used to compare the allelic and genotypic frequencies in the CAD and control groups with values predicted by Hardy-Weinberg equilibrium, and to compare the distribution of genotypes in the two SNPs with the severity of coronary lesions. The odds ratios (with the 95% confidence intervals) were calculated by logistic regression analysis. Two-tailed p*-*values < 0.05 indicated significance. All statistical analyses were done with SPSS version 15.0 software (SPSS, Inc, Chicago).

## Results

###  Baseline characteristics of the control and CAD groups

Patients with CAD had a greater average age and higher concentrations of insulin, TG and apo B when compared with the controls (p < 0.05-0.01) (Table 1). The prevalence of hypertension and T2DM was significantly higher in CAD patients than in the controls (p < 0.05). In contrast, the two groups did not differ significantly in their mean values for BMI, FBS, TC, LDL-C, HDL-C and apo A1, or in their sex ratio, proportion of smokers, and family history of cardiovascular diseases (CVD).

###  Polymorphism frequency and relationship to gender

Three genotypes were detected in rs822395 (AA, AC and CC) and rs266729 (CC, CG and GG). There was no deviation from Hardy-Weinberg equilibrium in the control group, nor were there significant differences in the frequencies of genotypes or alleles for these two SNPs between the control and CAD groups (Table 2).

Table 3 shows the frequency of polymorphisms according to gender. There were no significant differences in the distribution of genotypes AA, AC and CC at rs822395 between women of the two groups (p = 0.27). In contrast, the frequencies of alleles C and G at rs266729 showed borderline significance (p = 0.07) between the control and CAD groups and female carriers of allele G at rs266729 had a higher risk of CAD than allele C carriers (OR = 1.30, 95% CI: 1.09-2.64, p = 0.02). There were no significant differences in the distribution of these two SNPs among males of the control and CAD groups (data not shown).

###  Distribution of genotypes for the two SNPs in relation to the severity of coronary lesions

There were no significant differences in the frequencies of polymorphisms for the two SNPs among CAD patients with one, two or three stenosed vessels (p = 0.77 and p = 0.53 for rs822395 and rs266729, respectively) (data not shown). These findings indicate that there was no relationship between genotype and the severity of coronary lesions.

## Discussion

The results of this investigation failed to demonstrate an association between SNPs rs822395 and rs266729 of the adiponectin gene and CAD in a Chinese population, although the frequencies of alleles C and G at rs266729 in females showed borderline significance between the control and CAD groups, and female carriers of allele G at rs266729 had a higher risk of CAD compared with allele C carriers.

Adiponectin is a 244-amino acid protein that is secreted exclusively by adipocytes. This protein has been proposed to protect against CVD via its metabolic anti-inflammatory effects mediated by cross-talk between the cAMP-PKA and NF-kappaB signaling pathways (Ouchi *et al.*, 2000), by down-regulating adhesion molecule expression on endothelial cells (Ouchi *et al.*, 1999), and by acting in the homeostatic control of glucose, lipid, and energy metabolism with consequent lipid clearance and reversal of cardiovascular risk factors (Matsubara *et al.*, 2002; Cnop *et al.*, 2003; Ryo *et al.*, 2004; Trujillo and Scherer, 2005; Kawano and Arora, 2009). Adiponectin also stimulates fatty acid oxidation, decreases plasma TG and improves glucose metabolism by increasing insulin sensitivity (Haluzík *et al.*, 2004; Kawano and Arora, 2009). However, the lack of consistent data on the association between adiponectin, its genetic variants and the risk of CAD means that the precise role of this protein in CAD and CVD in general is still unclear (Antoniades *et al.*, 2009).

Although the adiponectin gene has been considered a candidate gene for CAD, Pischon *et al.* (2007) recently showed that of five common SNPs for this gene (rs266729, -11365C > G; rs822395, -4034A > C; rs822396, -3964A > G; rs2241766, +45T > G; and rs1501299, +276G > T) only the -4034CC genotype was related to an increased risk of nonfatal myocardial infarction or fatal CAD compared with the AA genotype in women [relative risk (RR): men, 1.69; 95% CI: 0.99-2.89; women, 2.04; 95% CI: 1.20-3.49]; other SNPs or haplotypes defined by the five SNPs were not consistently related to a risk of CAD or to the plasma levels of adiponectin.

In the patients from eastern China studied here, there was no significant association between the SNPs rs822395 and rs266729 and the risk of CAD. However, female CAD patients had a higher frequency of allele G at rs266729 than control females, and female carriers of allele G at rs266729 had a greater risk of CAD compared with allele C carriers. These findings suggest that genetic factors may exert a greater influence in CAD in women than in men. Indeed, epidemiological data show that the risk of CAD differs between female and male patients as a result of hormonal differences and variable exposure to the risk factors involved in disease progression. This is the first report of a gender-specific relationship between the rs266729 variant of the adiponectin gene and the risk of CAD, and differs from the results reported by Pischon *et al.* (2007). The reasons for this discrepancy are unclear, although ethnic and geographic differences in the populations studied could partly account for these variations. The CAD patients studied here had a greater prevalence of hypertension and T2DM, higher levels of insulin, TG and apo B, and a greater mean age than the controls.

This study has some limitations that could influence the interpretation of our results. First, the study cohort was obtained from a single hospital in eastern China so it is unlikely that the subjects adequately represented the characteristics of populations included in other studies. However, the well-designed study protocol and methods used here, together with the fact that all of the participants enrolled in the CAD and non-CAD groups underwent elective CAG meant that our results were reliable for the population studied. Second, since we did not measure the circulating adiponectin levels it was not possible to assess the effect of genetic variants of adiponectin on protein production – the limited data available in the literature indicate no consistent relationship between adiponectin variants and the plasma concentrations of this protein. Third, since CAD is produced by a variety of genes and environmental factors, various other genes could potentially contribute to the overall phenotype of CAD. This situation is further complicated by the fact that some risk factors for CAD, *e.g.*, certain genotypes and alleles, may be specific for particular ethnic and/or geographic populations. We therefore cannot exclude the possibility that gene-gene and/or gene-environment interactions may have influenced the results obtained here.

## Figures and Tables

**Table 1:** Baseline characteristics of the two groups studied.

	Control	CAD
Number of individuals	237	198
Gender – male (%)	109 (46.0)	107 (54.0)
Age (years)	54.5 ± 10.2	60.6 ± 10.1^†^
Hypertension (%)	129 (54.4)	130 (65.7)^*^
Type 2 diabetes mellitus (%)	29 (12.2)	42 (21.2)^*^
Smokers (%)	58 (24.5)	63 (31.8)
Family history of CVD (%)	76 (32.1)	75 (37.9)
BMI (kg/m^2^)	24.40 ± 4.35	24.96 ± 4.35
Fasting blood glucose (mmol/L)	5.55 ± 1.34	5.78 ± 1.60
Insulin (mU/L)	12.83 ± 10.17	15.51 ± 10.80^*^
TC (mmol/L)	4.47 ± 0.92	4.67 ± 0.91
TG (mmol/L)	1.51 ± 0.94	1.80 ± 0.93^†^
LDL-C (mmol/L)	2.74 ± 0.74	2.82 ± 0.76
HDL-C (mmol/L)	1.16 ± 0.26	1.16 ± 0.28
Apo A1 (g/L)	1.17 ± 0.23	1.15 ± 0.21
Apo B (g/L)	0.84 ± 0.27	0.92 ± 0.28^*^

Data are expressed as the number of individuals (percentage in parentheses) or the mean ± SD, as appropriate. ^*^p < 0.05 and ^†^p < 0.01 *vs.* control group. apo A1, apolipoprotein A1; apo B, apolipoprotein B; BMI, body mass index; CAD, coronary artery disease; CVD, cardiovascular disease; HDL-C, high-density lipoprotein-cholesterol; LDL-C, low-density lipoprotein-cholesterol; TC, total cholesterol; TG, triglyceride.

**Table 2:** Genotype and allele distributions for rs822395 and rs266729 in control and CAD groups.

	Control (%)	CAD (%)	OR (95% CI)
rs822395			
AA	175 (73.8)*	143 (72.2)*	
AC	59 (24.9)	48 (24.2)	1.00 (0.64-1.55)
CC	3 (1.3)	7 (3.5)	2.86 (0.73-11.24)
p	0.29		
Relative allele frequencies			
Allele A	409 (86.3)	334 (84.3)	
Allele C	65 (13.7)	62 (157)	1.17 (0.80-1.7)
p	0.44		
rs266729			
CC	146 (61.6)	110 (55.6)	
CG	76 (32.1)	72 (36.4)	1.26 (0.84-1.89)
GG	15 (6.3)	16 (8.1)	1.42 (0.67-2.99)
p	0.42		
Relative allele frequencies			
Allele C	368 (77.6)	292 (73.7)	
Allele G	106 (22.4)	104 (26.3)	1.24 (0.91-1.69)
p	0.20		

*Number of individuals with percentage in parentheses. CAD – coronary artery disease, OR – odds ratio, 95% CI - 95% confidence interval. The chi-square test and likelihood ratio test were used to analyze the genotypes and alleles, respectively. p is the level of significance for the CAD group compared to the control group.

**Table 3:** Genotype and allele distributions for rs822395 and rs266729 among women in the control and CAD groups.

	Control (%)	CAD (%)	OR (95% CI)
rs822395			
AA	97 (75.8)	72 (79.1)	
AC	29 (22.0)	15 (16.5)	0.70 (0.35-1.40)
CC	2 (1.6)	4 (4.4)	2.69 (0.48-15.12)
p	0.27		
Relative allele frequencies			
Allele A	223 (87.1)	159 (87.4)	
Allele C	33 (12.9)	23 (12.6)	0.98 (0.55-1.73)
p	0.94		
rs266729			
CC	84 (65.6)	46 (50.5)	
CG	37 (28.9)	36 (39.6)	1.78 (0.99-3.18)
GG	7 (5.5)	9 (9.9)	2.35 (0.82-6.72)
p	0.07		
Relative allele frequencies			
Allele C	205 (80.1)	128 (70.3)	
Allele G	51 (19.9)	54 (29.7)	1.30 (1.09-2.64)
p	0.02		

See Table 2 for abbreviations.
